# Supplementary material for: An open-source framework for large-scale, flexible evaluation of biomedical text mining systems
Source: J Biomed Discov Collab. 2008 Jan 29;3:1. doi: 10.1186/1747-5333-3-1 (PMC2276192; doi:10.1186/1747-5333-3-1)
Supplement: Additional file 1 — Evaluation platform source code. The additional file contains the software evaluation framework discussed in this paper. The most current release can be downloaded from SourceForge.net [1]. See the accompanying README file for instructions on installation and use. Also included in the distribution is a collection of UIMA wrappers for some commonly used BioNLP tools and annotated corpora. [file 1747-5333-3-1-S1.zip › bionlp_uima_repository-1.0.1/docs/api/src-html/edu/uchsc/ccp/util/nlp/stemmer/PorterStemmer_Util.html]

```
001    package edu.uchsc.ccp.util.nlp.stemmer;
002    
003    import org.tartarus.PorterStemmer;
004    
005    /**
006     * Extends the original Porter Stemmer implementation by Martin Porter (See {@link PorterStemmer}) to accept <code>String</code> input.
007     * 
008     * @author William A Baumgartner, Jr.
009     * 
010     */
011    public class PorterStemmer_Util extends PorterStemmer {
012    
013        /**
014         * Stems the input text
015         * 
016         * @param inputText
017         *            text to stem
018         * @return the stemmed text using the Porter algorithm
019         */
020        public String stem(String inputText) {
021            inputText = inputText.toLowerCase();
022            add(inputText.toCharArray(), inputText.toCharArray().length);
023            stem();
024            return toString();
025        }
026    
027    }
```
